# The GEnetic Syntax Score: a genetic risk assessment implementation tool grading the complexity of coronary artery disease—rationale and design of the GESS study

**DOI:** 10.1186/s12872-021-02092-5

**Published:** 2021-06-08

**Authors:** Ioannis S. Vizirianakis, Fani Chatzopoulou, Andreas S. Papazoglou, Efstratios Karagiannidis, Georgios Sofidis, Nikolaos Stalikas, Christos Stefopoulos, Konstantinos A. Kyritsis, Nikolaos Mittas, Nikoleta F. Theodoroula, Aggeliki Lampri, Eleni Mezarli, Anastasios Kartas, Dimitrios Chatzidimitriou, Anna Pappa-Konidari, Eleftherios Angelis, Ηaralambos Karvounis, Georgios Sianos

**Affiliations:** 1 Laboratory of Pharmacology, School of Pharmacy, Aristotle University of Thessaloniki, Thessaloniki, Greece; 2 Department of Life and Health Sciences, University of Nicosia, 1700 Nicosia, Cyprus; 3 Laboratory of Microbiology, School of Medicine, Aristotle University of Thessaloniki, Thessaloniki, Greece; 4 Labnet Laboratories, Thessaloniki, Greece; 5 Department of Cardiology, AHEPA University Hospital, Aristotle University of Thessaloniki, St. Kiriakidi 1, 54636 Thessaloniki, Greece; 6 Department of Chemistry, International Hellenic University, Kavala, Greece; 7 Department of Informatics, Aristotle University of Thessaloniki, Thessaloniki, Greece

**Keywords:** Genetics, Pharmacogenomics, SNPs, NGS, Biomarkers, Coronary artery disease, SYNTAX score, Acute coronary syndrome

## Abstract

**Background:**

Coronary artery disease (CAD) remains one of the leading causes of mortality worldwide and is associated with multiple inherited and environmental risk factors. This study is designed to identify, design, and develop a panel of genetic markers that combined with clinical and angiographic information, will facilitate the creation of a personalized risk prediction algorithm (GEnetic Syntax Score—GESS). GESS score could be a reliable tool for predicting cardiovascular risk for future adverse events and for guiding therapeutic strategies.

**Methods:**

GESS (ClinicalTrials.gov Identifier: NCT03150680) is a prospective, non-interventional clinical study designed to enroll 1080 consecutive patients with no prior history of coronary revascularization procedure, who undergo scheduled or emergency coronary angiography in AHEPA, University General Hospital of Thessaloniki. Next generation sequencing (NGS) technology will be used to genotype specific single-nucleotide polymorphisms (SNPs) across the genome of study participants, which were identified as clinically relevant to CAD after extensive bioinformatic analysis of literature-based SNPs. Enrichment analyses of Gene Ontology-Molecular Function, Reactome Pathways and Disease Ontology terms were also performed to identify the top 15 statistically significant terms and pathways. Furthermore, the SYNTAX score will be calculated for the assessment of CAD severity of all patients based on their angiographic findings. All patients will be followed-up for one-year, in order to record any major adverse cardiovascular events.

**Discussion:**

A group of 228 SNPs was identified through bioinformatic and pharmacogenomic analysis to be involved in CAD through a wide range of pathways and was correlated with various laboratory and clinical parameters, along with the patients' response to clopidogrel and statin therapy. The annotation of these SNPs revealed 127 genes being affected by the presence of one or more SNPs. The first patient was enrolled in the study in February 2019 and enrollment is expected to be completed until June 2021. Hence, GESS is the first trial to date aspiring to develop a novel risk prediction algorithm, the GEnetic Syntax Score, able to identify patients at high risk for complex CAD based on their molecular signature profile and ultimately promote pharmacogenomics and precision medicine in routine clinical settings.

*Trial registration* GESS trial registration: ClinicalTrials.gov Number: NCT03150680. Registered 12 May 2017- Prospectively registered, https://clinicaltrials.gov/ct2/show/NCT03150680.

## Background

Coronary artery disease (CAD) is a complex, multifactorial disease driven by the cumulative and interactive modular effects of gene–gene, gene–environment and epigenetic interactions [1]. Notwithstanding intense investigation in the postgenomic era, the fundamental biological pathways underlying the multidecade process of atherosclerotic formation and chronic inflammation in CAD have not yet been addressed [2]. The need for unraveling the molecular and genetic underpinnings of CAD at a deeper level is stressed nowadays, due to exceptionally high mortality rates of CAD, despite the expanded arsenal of precision medicine [3]. Therefore, defining CAD will enable the treatment of patients on the basis of a better understanding of their clinical presentations.

The potential for genotype-guided precision medicine is pointed out by recently emerging evidence from large scale studies investigating various gene expressions in patients with CAD. Hitherto, several Genome-Wide Association Studies (GWAS) have mapped more than 150 single-nucleotide polymorphisms (SNPs) potently implicated in CAD pathogenesis [1, 4–7]. These candidate variants are not yet established though and as Next Generation Sequencing (NGS) becomes the heart of high-throughput genotyping technologies, several plausible genetic variants linked with multifactorial traits of CAD might be discovered, shedding light on the road of personalized medicine [8]. Meanwhile, significant therapeutic implications emerge from the integration of genetic data into predictive risk scores. Specifically, several studies have been envisaged, in order to correlate distinct genetic variants with modulation of the risk for CAD occurrence or progression [9–11]. In those studies the severity of CAD has been assessed via clinical, laboratory or imaging parameters, but not with the Synergy Between Percutaneous Coronary Intervention With Taxus and Coronary Artery Bypass Graft Surgery (SYNTAX) score yet [12–15].

The SYNTAX score is the best-known scoring algorithm to evaluate CAD complexity as a comprehensive angiographic grading tool taking into consideration anatomic risk factors [16]. According to the extent of CAD, this score facilitates the objective guidance of decision-making between coronary artery bypass grafting (CABG) surgery and percutaneous coronary intervention (PCI). Despite, the SYNTAX score relies on invasive coronary angiography findings and the discovery of risk stratification algorithms that facilitate non-invasive estimation of CAD complexity could alter the prognostic plan in patients with CAD.

The rationale behind this prospective study is to associate, for the first time, the severity of CAD, as assessed by the SYNTAX score, with patients’ genomic profile in a real-world setting of patients undergoing coronary angiography [16]. The desirable goal is to corroborate genomic and pharmacogenetic research on CAD exploring the potential clinical association of 228 selected SNPs with CAD and individualized response to clopidogrel and statin therapy, which could disentangle gene expression alterations in blood of patients with CAD. Ultimately, the GESS trial aspires to develop a genetic SYNTAX score that could non-invasively enable the identification of patients with complex and severe CAD after a blood-based gene expression analysis. This study is designed to contribute to recent calls for implementing genotype-guided precision medicine decisions, by aiding the clinicians to achieve improved prediction and therapy outcomes for CAD patients [3].

## Methods

### Study design and population

GESS (ClinicalTrials.gov Identifier: NCT03150680) is an ongoing prospective, single‐center, cohort study enrolling patients undergoing coronary angiography.

Ethical approval was obtained from the Scientific Committee of AHEPA University Hospital (reference number 309/11–05-2017). Written informed consent will be obtained from each patient prior to study enrollment and the trial procedures conform with the Declaration of Helsinki [17].

GESS study is designed to enroll 1080 consecutive adult patients admitted to AHEPA University Hospital of Thessaloniki, Greece and undergoing coronary angiography for clinical purposes. Coronary angiography can be performed either on an emergency basis or scheduled. For the purpose of this research, patients with history of prior percutaneous coronary intervention or coronary artery bypass grafting and patients unwilling to provide informed consent will be excluded from the study. The selection criteria of the study are presented in detail in Table 1.

**Table 1:** Eligibility criteria for the enrollment in the GESS study

Inclusion criteria	Exclusion criteria
Patients undergoing coronary angiography	Medical history of prior coronary revascularization procedure
Age > 18 years old	Cardiopulmonary arrest at admission
Informed consent for study participation	Severe concurrent disease with life expectancy less than 12 months

Pre-specified clinical data, including demographic characteristics, medical history, medication and clinical presentation will be recorded for the entire study population by research study coordinators under standardized methods. Accordingly, study participants will be classified into 3 main subsets, based on their clinical presentation: 1. patients undergoing preoperative coronary angiography without symptoms suggestive of CAD, 2. patients with chronic coronary syndrome, and iii. patients with acute coronary syndrome.

Moreover, all enrolled patients will undergo selective coronary angiography, which will be performed through radial or femoral artery approach in the cardiac catheterization laboratory of the hospital. Images obtained will be assessed by experienced interventionalists (GS1, GS2), blinded to the study protocol, who will be in charge of calculating the SYNTAX scores. According to their SYNTAX score, patients will be categorized into the following groups: i. low SYNTAX score (0–22) group, ii. intermediate SYNTAX score (23–32) group, and iii. high SYNTAX score (> 32) group [16].

Additionally, peripheral blood samples will be drawn on the enrollment day- prior to coronary angiography—for genomic profiling. The vials of drawn blood will be aliquoted and stored as whole blood, plasma, serum, and buffy coat.

The first participant of the study was enrolled in February 2019 and 783 patients have been recruited through November 2020. Completion of patient enrollment is expected until June 2021.

Telephone follow-up will be systematically carried out for every study subject at 1 year after enrollment, in order to document the incidence of CAD symptoms, major adverse cardiovascular and cerebrovascular events (MACCE-need for coronary revascularization, myocardial infarction, stroke/ transient ischemic attack or all-cause mortality) and bleeding complications (Bleeding Academic Research Consortium classification score [18]).

### Genotyping and bioinformatic analysis

Peripheral whole blood will be collected and labeled with a unique barcode to ensure anonymization and unbiased assessment. High quality genomic DNA will be extracted using commercial kits (Qiagen) and will be quantified by spectrophotometry using Nanodrop 1000 (Thermo Fisher). Ultrasensitive targeted NGS of extracted DNA (40 ng) will be performed using custom QIAseq Targeted DNA Panel (Qiagen) containing primers for the enrichment of the 228 SNPs of interest. The produced molecularly barcoded libraries will be quantified by Qubit 3 Fluorometer (Invitrogen) and real time PCR (QIAseq Library Quant Assay kit). Sequencing will be performed by sequencing by synthesis (SBS) chemistry on MiniSeq Platform of Illumina using the MiniSeq Mid Output Kit (300-cycles). The generated NGS data (in fastq format) will be analyzed with the CLC Genomics Workbench (Qiagen) bioinformatics software and the genotype of each SNP will be determined.

### Biostatistics and disease ontology enrichment analysis

**Fig. 1:**
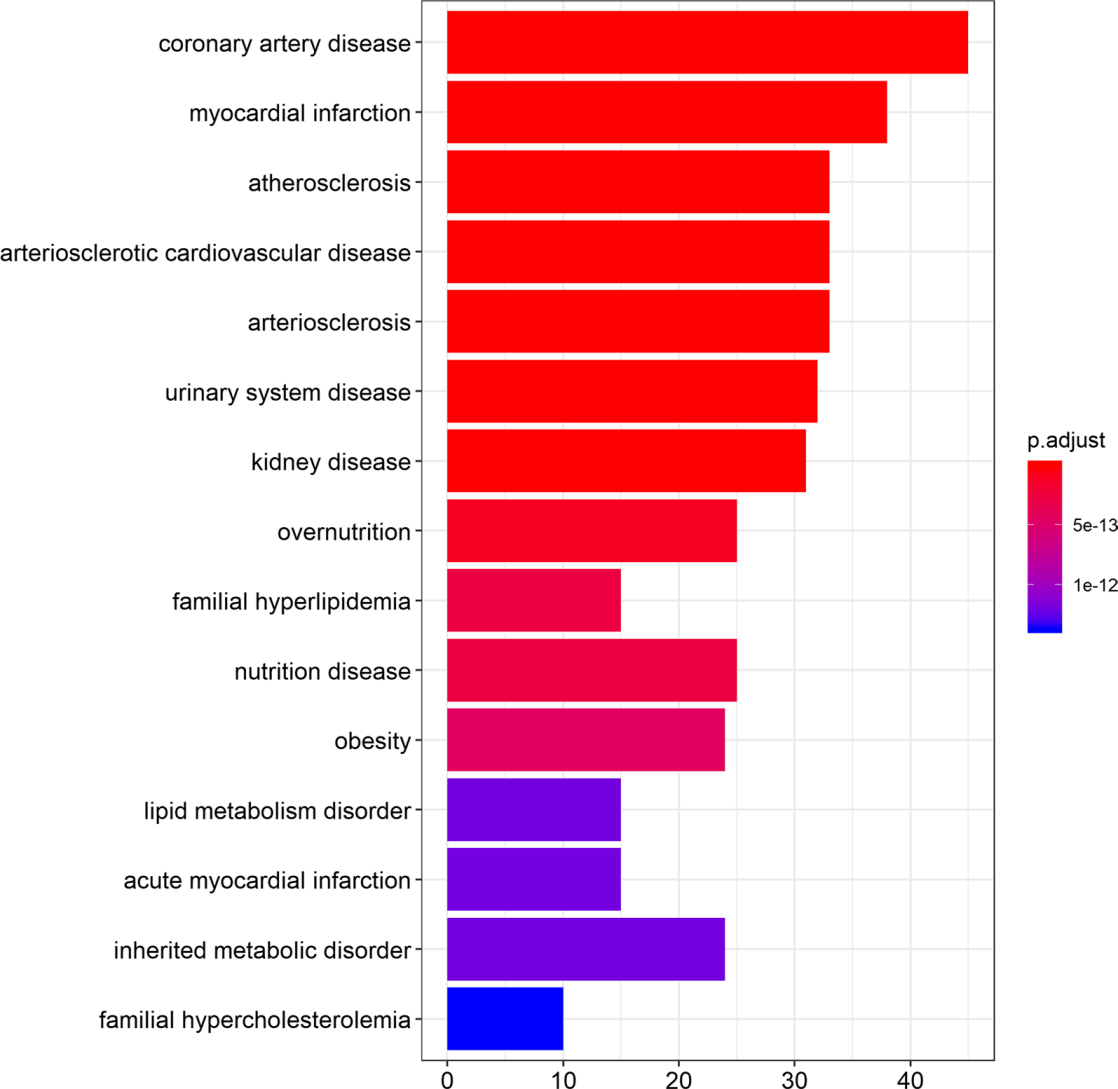
Barplot of top 15 statistically significant disease ontology terms. Disease terms and number of genes are displayed in y and x axis, respectively. Enrichment analysis and barplot creation were performed using clusterProfiler and DOSE

**Fig. 2:**
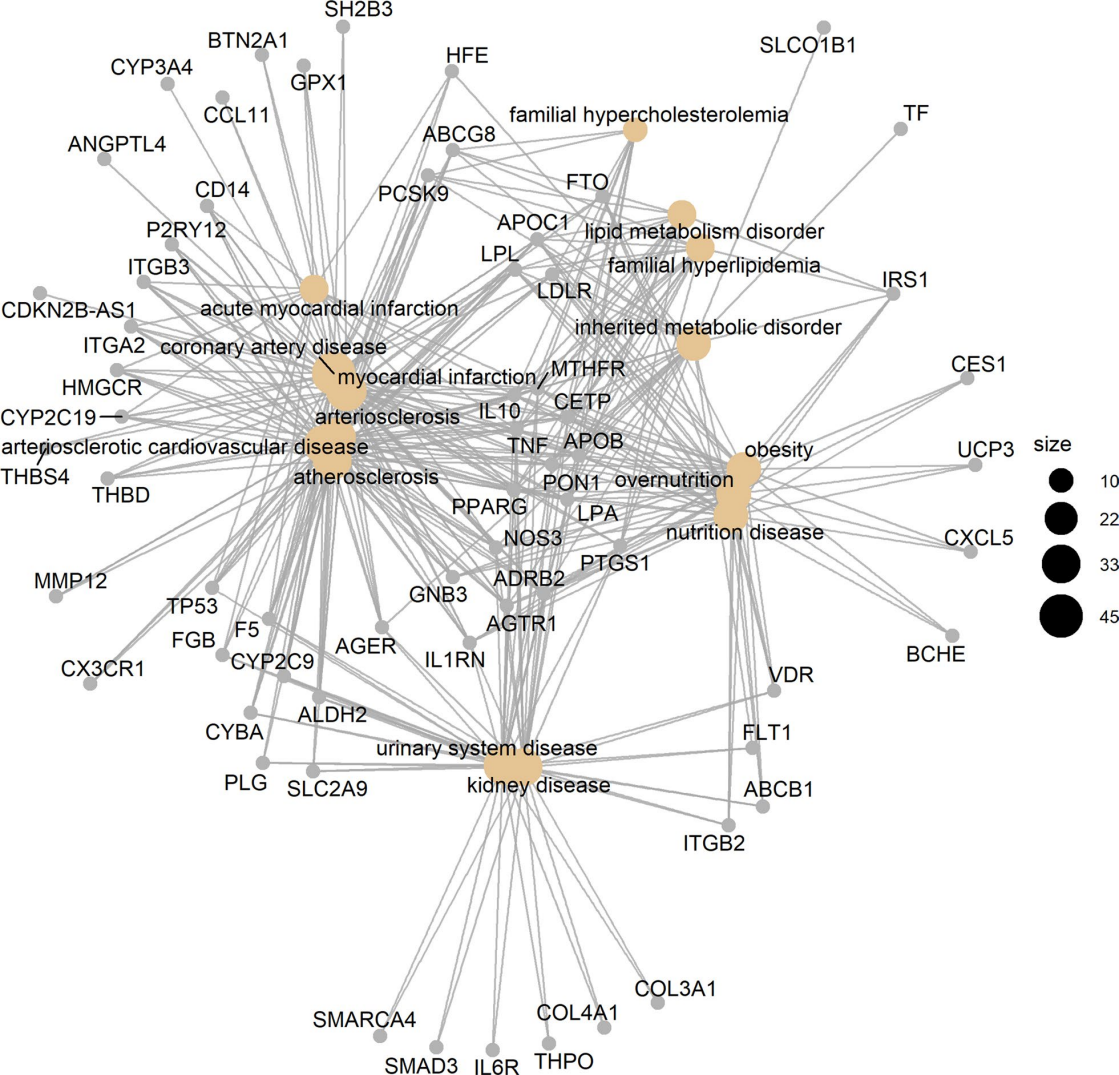
Gene concept network of top 15 statistically significant disease ontology terms. Enrichment analysis and network creation were performed using clusterProfiler and DOSE

We sought to identify genes whose coding sequence and/or expression levels are affected by the selected 228 SNPs studied here. To this end, data mining was performed from dbSNP database using reutils [19, 20]. In addition, further information on genes, associated with the selected SNPs through GWAS, were retrieved from HumanMine database [21] using InterMineR [22]. Our approach led to the formation of a list with 127 genes that have been associated with the selected SNPs. Next, enrichment analysis was performed to identify statistically significant disease terms, whose involved genes are overrepresented in our gene list. Enrichment analysis, with Benjamini–Hochberg adjusted *p-value* < 0.001, was performed using clusterProfiler [23] and DOSE [24] (Figs. 1 and 2).

### Statistical considerations

#### Sample size estimation and endpoints of the study

The primary endpoint of the study is to discover potential correlations of the SYNTAX score with patients' genomic profile and create a blood-based gene expression test (genetic SYNTAX score) which could accurately identify patients at high risk for CAD of moderate or high severity. For the estimation of the sample size the G*Power [25, 26] and Epi Info (StatCalc) [27] software tools were used. To this regard, we made use of the exact sampling distribution of the squared multiple correlation coefficient implemented in G*Power assuming 250 predictors, a two-tailed test, power of 0.9, significance level of 0.05, ρ^2^ = 0.13 and a ratio of unexposed to exposed equal to 2 (based on a pilot study on 100 patients). The initial sample size was finally increased by 10% because of the possibility that some patients might be lost to follow-up. Hence, we aim for a total sample of 1080 patients.

Secondary endpoints of the study are the development of a panel of genetic markers that, in conjunction with clinical parameters, could strongly predict the occurrence of MACCE or any bleeding events during follow up.

### Statistical analysis

Descriptive analysis will be used to summarize the data. Specifically, results will contain statistics as mean, standard deviation, median, minimum and maximum values, whereas for categorical variables the frequency distribution tables with number of cases and percentage distribution will be presented. Statistical hypothesis testing procedures (*Kolmogorov–Smirnov* and *Shapiro–Wilk*) will be conducted for continuous variables to check, whether they satisfy the normality assumption. Given the fact that the response variable (SYNTAX score) presents a heavily-skewed and non-normal distribution with an excess number of zeros, non-parametric statistical hypothesis tests will be used for the investigation of the main effects of categorical variables on the population median values of the response variable. More specifically, the *Mann–Whitney* and *Kruskal–Wallis* followed by pair-wise comparisons through Mann–Whitney test using *Bonferroni’s correction* will be conducted. The investigation of the relationship between SYNTAX score and the set of continuous variables will be performed using the non-parametric *Spearman’s correlation coefficient*.

The model building process will be based on *Hurdle Models* that are a class of modeling techniques able to handle excess zeros and overdispersion of SYNTAX score variable. Describing briefly, the *Hurdle Model* has two parts: (*i*) a *zero hurdle part* which models the right-censored outcome SYNTAX score variable indicating patients with a zero-count ($Y = 0$) or patients with a positive count ($Y = 1$), where all values larger than zero are censored (i.e. fixed at one) and (*ii*) a *truncated count part* modeling the total number of SYNTAX score for patients presenting a non-zero count ($Y > 0$). Regarding the identification of the best set of predictors for each part of the model, a feature selection search strategy based on *Akaike Information Criterion* will be utilized, in which the set of predictors are included in the *full model* and at each step of the iterative process, a predictor is dropped out. To assess the fitting performance of the final model, well-known evaluation metrics for regression (e.g. mean and median squared, absolute and percentage errors) and classification tasks (*accuracy*, *F*-*measure*, *G*-*mean*, *precision* and *recall*) will be used, whereas for the evaluation of the prediction performance of the model, data-generating schemas (i.e. *holdout* and *k-fold cross-validation*) that split the available dataset into training and test sets will be performed. In addition, graphical evaluation of model’s performance will be assessed through appropriate visualization methods, such as *Receiver Operating Characteristic* (ROC) and *Precision-Recall* curves for the zero-hurdle part and *Regression Error Characteristic* (REC) curves for the truncated count part.

*Survival analysis* methods will be also performed for examining patients at follow-up period. More specifically, the non-parametric *Kaplan–Meier* analysis will be conducted for graphically evaluating the survival function of patients, while *log-rank tests* will be conducted for investigating effects of different factors on survival distribution. Finally, *Cox Regression* analysis will be performed to build a multivariate regression model between several predictors and the survival time of patients.. Statistical analysis will be performed via the R statistical programming language. In all tests a difference will be considered as statistically significant when p-value (significance) will be less than 0.05, while all conducted tests will be two-tailed (non-directional).

## Discussion

GESS is a prospective ongoing study designed to determine the impact of the presence of several genetic variants on CAD severity. The aim of this study is to further understand the pathogenesis of CAD by utilizing 3 fundamental pillars: (1) invasive coronary angiography and standardized SYNTAX score calculation; (2) revolutionary NGS technologies; and (3) systems biology-based bioinformatics. To our knowledge, hitherto, this is the first study designed to establish a prognostic blood assay for the association of the presence of a large number of SNPs with CAD severity, as evaluated via the SYNTAX score.

Endothelial dysfunction, oxidative stress and inflammation, which are the products of a multifactorial interplay between inherited and environmental risk factors, are established determinants of the atherosclerotic burden and CAD prognosis [4, 28]. Large GWAS have been conducted in order to locate CAD-associated variants (SNPs) and decipher the underlying genetic fundament of the disease [6, 29–35]. To date, a great number of susceptible multi-SNP loci have been identified with some of them reaching the stringent level of significance [6, 32, 34, 36, 37]. More specifically, more than 150 SNPs, in over 100 candidate genes have been annotated as CAD-relevant with specific loci, such as 9p21.3, 6q25.1, 2q36.3, showing the strongest association with disease phenotypic variance [5, 8, 38, 39]. The CARDIoGRAMplusC4D Consortium has carried out a meta-analysis in a total sample size of over 190.000 patients and demonstrated a highly significant correlation of 36 SNPs with CAD [6]. Furthermore, Liu et al. reported that the most studied multi-loci genes are those of angiotensin I converting enzyme, lipid and lipoprotein metabolism [1]. Hence, individual GWAS and meta-analyses have confirmed the speculated deterministic role of genetic predisposition in occurrence, progression of atherosclerosis and coronary plaque calcification, with multiple converging pathways, including cardiac muscle contraction, glycerolipid metabolism, and glycosaminoglycan biosynthesis [5, 32, 37, 40].

Nevertheless, GWAS have only provided population-attributable risk data and could not be transferred to an individual with CAD. During the last decade, the advent of NGS has enabled researchers to perform parallel analyses of hundreds of genes in an unbiased approach [8]. This is attracting widespread attention enhancing CAD translational study and aiding to close the gap between genotype and phenotype. In 2013 the CARDIoGRAMplusC4D Consortium reported that targeted sequencing with NGS can discover rare variants with high sensitivity, rendering NGS an essential genetics approach in the post-GWA study era [38].

Apart from genetic mapping, GWAS and NGS studies have also explored the clinical utility of genetic biomarkers for the creation of genetic risk scores [10, 11, 36, 41]. These algorithms would ideally predict the severity of CAD and the subsequent adverse outcomes aiming to identify patients with potential benefit from preventive care. For their development, researchers have examined the prognostic value of blood-based genetic panels, in comparison with imaging (myocardial perfusion imaging or coronary computed tomography angiography), angiographic (visual or quantitative assessment of coronary artery stenosis or Gensini) or clinical (GRACE) predictive scores [12, 15, 42–44]. COMPASS and PREDICT trials created 2 gene-expression scores outperforming clinical factors and non-invasive imaging in discriminating patients with > 50% stenosis [45, 46]. Despite, Labos et al. reported that the addition of their developed polygenic risk score to the GRACE risk score could not significantly improve risk classification in acute coronary syndrome admissions [42]. Moreover, weighted multi-locus risk scores have been created to predict recurrent vascular events or statin efficacy and atherosclerotic burden alterations in CAD populations [10, 47–49]. Nevertheless, limited data exist about the utility of genetic risk scores for the prediction of MACCE [11–13, 41].

To the best of our knowledge, GESS is the first study yet to investigate the association of such a large number of candidate SNPs (228) with SYNTAX-score-based CAD complexity. To this end, GESS emerges as a part of a research project aspiring to complement traditional risk factor assessment with panels of significant metabolomic and genomic biomarkers [50, 51]. The co-evaluation of novel risk factors and the complexity of CAD could significantly expand the concept of cardiovascular precision medicine.

Admittedly, the GESS trial is subject to some limitations that merit discussion. First, the single-center character of the study and the enrollment of patients from a Greek-based population may limit the generalizability of our findings, even if our sample will represent a broad spectrum of patients with CAD. Furthermore, patients of different age groups will comprise the study population, which might affect the rate of genetic influence in CAD severity, since the genetic component of variability is conceivably more common among younger individuals. Future studies should explore the combination of proposed genetic risk scores from multi-ethnic populations with panels of metabolomics, transcriptomics or proteomics, to achieve the desirable transition from “omics” to “panomics” [44]. Therefore, we could define CAD at the deepest level and clinical cardiologists would be guided in decision-making via an absolutely personalized approach.

## Conclusion

In conclusion, genotyping of patients presenting with CAD symptoms could potentially disentangle genetic risk variants implicated in CAD progression. The development of a panel with genetic markers combined with clinical and angiographic characteristics might contribute to implementing accurate risk stratification algorithms in CAD populations, with the potential to predict the emergence of CAD as well as the hazard for subsequent adverse events and modify therapeutic strategies. Besides that, the design of the study creates an interdisciplinary infrastructure that allows the clinical translation of molecular knowledge to guide decisions for individual and/or CAD patient groups. Importantly, such direction contributes to the establishment and application of processes that successfully implement genomics knowledge in the clinical setting within the concept of pharmacogenomics and precision medicine.

## Data Availability

Data are available from Georgios Sianos (e-mail: gsianos@auth.gr) upon reasonable request and with permission of AHEPA University Hospital.

## Abbreviations

CAD: Coronary artery disease
STEMI: ST elevation myocardial infarction
NSTEMI: Non-ST elevation myocardial infarction
MACCE: Major adverse cardiovascular and cerebrovascular events

## References

[1] Liu H, Liu W, Liao Y, et al. CADgene: a comprehensive database for coronary artery disease genes. *Nucleic Acids Res*. 2011;39(Database issue):D991–6. 10.1093/nar/gkq1106. 10.1093/nar/gkq1106 PMC3013698 21045063

[2] Vizirianakis IS (2004). Challenges in current drug delivery from the potential application of pharmacogenomics and personalized medicine in clinical practice. Curr Drug Deliv.

[3] Dainis AM, Ashley EA (2018). Cardiovascular precision medicine in the genomics era. JACC Basic Transl Sci.

[4] Prins BP, Lagou V, Asselbergs FW, Snieder H, Fu J (2012). Genetics of coronary artery disease: genome-wide association studies and beyond. Atherosclerosis.

[5] Burton PR, Clayton DG, Cardon LR, et al. The Wellcome Trust Case Control Consortium. Genome-wide association study of 14,000 cases of seven common diseases and 3,000 shared controls. *Nature*. 2007;447(7145):661–678. 10.1038/nature05911.Genome-wide. 10.1038/nature05911 PMC2719288 17554300

[6] Deloukas P, Kanoni S, Willenborg C (2013). Large-scale association analysis identifies new risk loci for coronary artery disease. Nat Genet.

[7] O’Donnell CJ, Kavousi M, Smith AV (2011). Genome-wide association study for coronary artery calcification with follow-up in myocardial infarction. Circulation.

[8] Kalayinia S, Goodarzynejad H, Maleki M, Mahdieh N (2018). Next generation sequencing applications for cardiovascular disease. Ann Med.

[9] Knowles JW, Zarafshar S, Pavlovic A (2017). Impact of a genetic risk score for coronary artery disease on reducing cardiovascular risk: a pilot randomized controlled study. Front Cardiovasc Med.

[10] Natarajan P, Young R, Stitziel NO (2017). Polygenic risk score identifies subgroup with higher burden of atherosclerosis and greater relative benefit from statin therapy in the primary prevention setting. Circulation.

[11] Zhao C, Zhu P, Shen Q, Jin L (2017). Prospective association of a genetic risk score with major adverse cardiovascular events in patients with coronary artery disease. Medicine (Baltimore).

[12] Thomas GS, Voros S, McPherson JA (2013). A blood-based gene expression test for obstructive coronary artery disease tested in symptomatic nondiabetic patients referred for myocardial perfusion imaging: The COMPASS study. Circ Cardiovasc Genet.

[13] Palmerini T, Calabrò P, Piscione F (2014). Impact of gene polymorphisms, platelet reactivity, and the SYNTAX score on 1-year clinical outcomes in patients with non-ST-segment elevation acute coronary syndrome undergoing percutaneous coronary intervention: the GEPRESS study. JACC Cardiovasc Interv.

[14] Kullo IJ, Jouni H, Austin EE (2016). Incorporating a genetic risk score into coronary heart disease risk estimates: effect on low-density lipoprotein cholesterol levels (the MI-GENES clinical trial). Circulation.

[15] Osadnik T, Strzelczyk JK, Lekston A (2016). The association of functional polymorphisms in genes encoding growth factors for endothelial cells and smooth muscle cells with the severity of coronary artery disease. BMC Cardiovasc Disord.

[16] Sianos G, Morel M-A, Kappetein AP (2005). The SYNTAX Score: an angiographic tool grading the complexity of coronary artery disease. EuroIntervention J Eur Collab Work Gr Interv Cardiol Eur Soc Cardiol.

[17] World Medical Association declaration of Helsinki (2013). Ethical principles for medical research involving human subjects. JAMA J Am Med Assoc.

[18] Mehran R, Rao SV, Bhatt DL (2011). Standardized bleeding definitions for cardiovascular clinical trials: a consensus report from the bleeding academic research consortium. Circulation.

[19] Sherry ST, Ward MH, Kholodov M (2001). dbSNP: the NCBI database of genetic variation. Nucleic Acids Res.

[20] Schöfl G. reutils: Talk to the NCBI EUtils. Published online 2016:1.

[21] Smith RN, Aleksic J, Butano D (2012). InterMine: a flexible data warehouse system for the integration and analysis of heterogeneous biological data. Bioinformatics.

[22] Kyritsis KA, Wang B, Sullivan J, Lyne R, Micklem G (2019). InterMineR: an R package for InterMine databases. Bioinformatics.

[23] Yu G, Wang L-G, Han Y, He Q-Y (2012). clusterProfiler: an R package for comparing biological themes among gene clusters. OMICS.

[24] Yu G, Wang L-G, Yan G-R, He Q-Y (2015). DOSE: an R/Bioconductor package for disease ontology semantic and enrichment analysis. Bioinformatics.

[25] Faul F, Erdfelder E, Lang A-G, Buchner A (2007). G*Power 3: a flexible statistical power analysis program for the social, behavioral, and biomedical sciences. Behav Res Methods.

[26] Faul F, Erdfelder E, Buchner A, Lang A-G (2009). Statistical power analyses using G*Power 3.1: tests for correlation and regression analyses. Behav Res Methods.

[27] Dean AG, Arner TG, Sunki GG, Friedman R, Lantinga M, Sangam S, Zubieta JC, Sullivan KM, Brendel KA, Gao Z, Fontaine N, Shu M, Fuller G, Smith DC, Nitschke DA and FR. Epi Info^TM^, a database and statistics program for public health professionals. *Centers Dis Control Prev Atlanta, Georg USA*. Published online 2011.

[28] Abraham G, Bhalala OG, De Bakker PIW, Ripatti S, Inouye M (2014). Towards a molecular systems model of coronary artery disease. Curr Cardiol Rep.

[29] Raina JK, Sharma M, Panjaliya RK, Dogra V, Bakaya A, Kumar P (2020). Association of ESR1 (rs2234693 and rs9340799), CETP (rs708272), MTHFR (rs1801133 and rs2274976) and MS (rs185087) polymorphisms with Coronary Artery Disease (CAD). BMC Cardiovasc Disord.

[30] Xu L-B, Zhang Y-Q, Zhang N-N (2020). Rs10757274 gene polymorphisms in coronary artery disease: a systematic review and a meta-analysis. Medicine (Baltimore).

[31] Wu Y, Wang W, Li X-Y (2019). Strong association between the interleukin-8-251A/T polymorphism and coronary artery disease risk. Medicine (Baltimore).

[32] The Wellcome Trust Case Control Consortium (2007). Genome-wide association study of 14,000 cases of seven common diseases and 3,000 shared controls. Nature.

[33] Erdmann J, Willenborg C, Nahrstaedt J (2011). Genome-wide association study identifies a new locus for coronary artery disease on chromosome 10p.1123. Eur Heart J.

[34] Larson MG, Atwood LD, Benjamin EJ, et al. Framingham Heart Study 100K project: genome-wide associations for cardiovascular disease outcomes. *BMC Med Genet*. 2007;8 Suppl 1(Suppl 1):S5. 10.1186/1471-2350-8-S1-S5. 10.1186/1471-2350-8-S1-S5 PMC1995607 17903304

[35] van Iperen EPA, Sivapalaratnam S, Holmes MV, Hovingh GK, Zwinderman AH, Asselbergs FW (2016). Genetic analysis of emerging risk factors in coronary artery disease. Atherosclerosis.

[36] Khera AV, Emdin CA, Drake I (2016). Genetic risk, adherence to a healthy lifestyle, and coronary disease. N Engl J Med.

[37] Samani NJ, Erdmann J, Hall AS (2007). Genomewide association analysis of coronary artery disease. N Engl J Med.

[38] Shea J, Agarwala V, Philippakis AA (2011). Comparing strategies to fine-map the association of common SNPs at chromosome 9p21 with type 2 diabetes and myocardial infarction. Nat Genet.

[39] Hartiala JA, Han Y, Jia Q (2021). Genome-wide analysis identifies novel susceptibility loci for myocardial infarction. Eur Heart J.

[40] Zhao X, Luan Y-Z, Zuo X (2016). Identification of risk pathways and functional modules for coronary artery disease based on genome-wide SNP data. Genom Proteom Bioinf.

[41] Liu R, Song L, Jiang L (2020). Susceptible gene polymorphism in patients with three-vessel coronary artery disease. BMC Cardiovasc Disord.

[42] Labos C, Martinez SC, Leo Wang RH (2015). Utility of a genetic risk score to predict recurrent cardiovascular events 1 year after an acute coronary syndrome: a pooled analysis of the RISCA, PRAXY, and TRIUMPH cohorts. Atherosclerosis.

[43] Voros S, Rinehart S, Qian Z (2011). Prospective validation of standardized, 3-dimensional, quantitative coronary computed tomographic plaque measurements using radiofrequency backscatter intravascular ultrasound as reference standard in intermediate coronary arterial lesions. JACC Cardiovasc Interv.

[44] Voros S, Maurovich-Horvat P, Marvasty IB (2014). Precision phenotyping, panomics, and system-level bioinformatics to delineate complex biologies of atherosclerosis: rationale and design of the “Genetic Loci and the Burden of Atherosclerotic Lesions” study. J Cardiovasc Comput Tomogr.

[45] Giugliano RP, Ruff CT, Braunwald E (2013). Edoxaban versus warfarin in patients with atrial fibrillation. N Engl J Med.

[46] Rosenberg S, Elashoff MR, Beineke P (2010). Multicenter validation of the diagnostic accuracy of a blood-based gene expression test for assessing obstructive coronary artery disease in nondiabetic patients. Ann Intern Med.

[47] Mega JL, Stitziel NO, Smith JG (2015). Genetic risk, coronary heart disease events, and the clinical benefit of statin therapy: an analysis of primary and secondary prevention trials. Lancet (London, England).

[48] Erathi HV, Durgaprasad R, Velam V (2018). Evaluation of On-Clopidogrel platelet reactivity overtime, SYNTAX SCORE, genetic polymorphisms and their relationship to one year clinical outcomes in STEMI patients undergoing PCI. Minerva Cardioangiol.

[49] Tragante V, Doevendans PAFM, Nathoe HM (2013). The impact of susceptibility loci for coronary artery disease on other vascular domains and recurrence risk. Eur Heart J.

[50] Karagiannidis E, Papazoglou AS, Stalikas N (2021). Serum ceramides as prognostic biomarkers of large thrombus burden in patients with STEMI: a micro-computed tomography study. J Pers Med.

[51] E. Karagiannidis; G. Sofidis; A. S Papazoglou et al. Correlation of the severity of coronary artery disease with patients’ metabolic profile- rationale, design and baseline patient characteristics of the CorLipid trial. *BMC Cardiovasc Disord*. Published online 2021:1–7. 10.1186/s12872-021-01865-2. 10.1186/s12872-021-01865-2 PMC7869241 33557756

