# Correction to: The GEnetic Syntax Score: a genetic risk assessment implementation tool grading the complexity of coronary artery disease—rationale and design of the GESS study

**DOI:** 10.1186/s12872-021-02122-2

**Published:** 2021-06-21

**Authors:** Ioannis S. Vizirianakis, Fani Chatzopoulou, Andreas S. Papazoglou, Efstratios Karagiannidis, Georgios Sofdis, Nikolaos Stalikas, Christos Stefopoulos, Konstantinos A. Kyritsis, Nikolaos Mittas, Nikoleta F. Theodoroula, Aggeliki Lampri, Eleni Mezarli, Anastasios Kartas, Dimitrios Chatzidimitriou, Anna Papa-Konidari, Eleftherios Angelis, Ηaralambos Karvounis, Georgios Sianos

**Affiliations:** 1 Laboratory of Pharmacology, School of Pharmacy, Aristotle University of Thessaloniki, Thessaloniki, Greece; 2 Department of Life and Health Sciences, University of Nicosia, 1700 Nicosia, Cyprus; 3 Laboratory of Microbiology, School of Medicine, Aristotle University of Thessaloniki, Thessaloniki, Greece; 4 Labnet Laboratories, Thessaloniki, Greece; 5 Department of Cardiology, AHEPA University Hospital, Aristotle University of Thessaloniki, St. Kiriakidi 1, 54636 Thessaloniki, Greece; 6 Department of Chemistry, International Hellenic University, Kavala, Greece; 7 Department of Informatics, Aristotle University of Thessaloniki, Thessaloniki, Greece

## Correction to: BMC Cardiovasc Disord (2021) 21:284 https://doi.org/10.1186/s12872-021-02092-5

Following publication of the original article [1], the authors identified an error in the author name of Anna Papa-Konidari.

The incorrect author name is: Anna Pappa-Konidari.

The correct author name is: Anna Papa-Konidari.

The original article has been corrected as well.
